# Evidence of Illegitimate Recombination Between Two *Pasteurellaceae* Plasmids Resulting in a Novel Multi-Resistance Replicon, pM3362MDR, in *Actinobacillus pleuropneumoniae*

**DOI:** 10.3389/fmicb.2018.02489

**Published:** 2018-10-23

**Authors:** Yinghui Li, Giarlã Cunha da Silva, Yanwen Li, Ciro C. Rossi, Roberto Fernandez Crespo, Susanna M. Williamson, Paul R. Langford, Denise Mara Soares Bazzolli, Janine T. Bossé

**Affiliations:** ^1^Section of Paediatrics, Department of Medicine, Imperial College London, London, United Kingdom; ^2^Shenzhen Center for Disease Control and Prevention, Shenzhen, China; ^3^Laboratório de Genética Molecular de Bactérias, Departamento de Microbiologia, Instituto de Biotecnologia Aplicada à Agropecuária, Universidade Federal de Viçosa, Viçosa, Brazil; ^4^Animal and Plant Health Agency, Addlestone, United Kingdom

**Keywords:** plasmids, antimicrobial resistance, tetracycline, respiratory tract, *Pasteurellaceae*

## Abstract

Evidence of plasmids carrying the tetracycline resistance gene, *tet*(B), was found in the previously reported whole genome sequences of 14 United Kingdom, and 4 Brazilian, isolates of *Actinobacillus pleuropneumoniae.* Isolation and sequencing of selected plasmids, combined with comparative sequence analysis, indicated that the four Brazilian isolates all harbor plasmids that are nearly identical to pB1001, a plasmid previously found in *Pasteurella multocida* isolates from Spain. Of the United Kingdom isolates, 13/14 harbor plasmids that are (almost) identical to pTetHS016 from *Haemophilus parasuis*. The remaining United Kingdom isolate, MIDG3362, harbors a 12666 bp plasmid that shares extensive regions of similarity with pOV from *P. multocida* (which carries *bla_ROB-1_*, *sul2*, and *strAB* genes), as well as with pTetHS016. The newly identified multi-resistance plasmid, pM3362MDR, appears to have arisen through illegitimate recombination of pTetHS016 into the stop codon of the truncated *strB* gene in a pOV-like plasmid. All of the *tet*(B)-carrying plasmids studied were capable of replicating in *Escherichia coli*, and predicted origins of replication were identified. A putative origin of transfer (*oriT*) sequence with similar secondary structure and a *nic*-site almost identical to that of RP4 was also identified in these plasmids, however, attempts to mobilize them from an RP4-encoding *E. coli* donor strain were not successful, indicating that specific conjugation machinery may be required.

## Introduction

Resistance to tetracycline is widespread amongst isolates of *Actinobacillus pleuropneumoniae* in many countries (Archambault et al., 2012; Vanni et al., 2012; Dayao et al., 2016; El Garch et al., 2016). Despite this, tetracyclines continue to be the most widely used antimicrobial for treatment of respiratory and other diseases in food-producing animals in the United Kingdom and other European countries (Borriello, 2013; Garcia-Migura et al., 2014; European Medicines Agency and European Surveillance of Veterinary Antimicrobial Consumption, 2017). Identification of the genes responsible for tetracycline resistance, and an understanding of the mechanisms underlying the spread of these genes, will help inform decisions regarding continued use of this important antimicrobial agent.

Although more than thirty different tetracycline resistance genes have been reported in different bacterial species (Chopra and Roberts, 2001), relatively few have been found in *A. pleuropneumoniae*, with *tet*(B) being the most common (Blanco et al., 2006; Dayao et al., 2016; Bossé et al., 2017; Michael et al., 2018). We recently reported that the *tet*(B) gene was found in the chromosome of 37.5% of United Kingdom isolates for which whole genome sequences (wgs) were determined, either as part of a large integrative conjugative element (ICE*Apl1*), or as a transposon insertion in the *comM* gene (Bossé et al., 2017). A further 14.5% of the tested isolates had *tet*(B) genes that appeared to be associated with plasmid sequences (Bossé et al., 2017). Wgs for six Brazilian isolates also indicate the presence of *tet*(B) associated with plasmid sequences in four of these isolates (Pereira et al., 2015). As small plasmids often appear to be common amongst members of the *Pasteurellaceae* (Michael et al., 2018), the aim of this study was to identify the *tet*(B) plasmids present in these sequenced isolates of *A. pleuropneumoniae*.

## Materials and Methods

### *A. pleuropneumoniae* Isolates and Plasmids

Information regarding the 14 United Kingdom and 4 Brazilian *A. pleuropneumoniae* isolates and their wgs data (Table 1) was compiled from details described previously (Pereira et al., 2015; Bossé et al., 2017). Data regarding the tetracycline resistance plasmids identified in this study are also shown (Table 1). Plasmid sequences were initially identified in the draft genome sequences using ResFinder (Zankari et al., 2012) to identify contigs containing the *tet*(B) gene. BLASTn was then used to identify sequences with the highest identity to each of these contigs. Where it appeared that a single contig matched a known plasmid, the ends of the contigs were analyzed to identify overlapping sequences that allowed closure into a circular plasmid. Where it appeared that the contigs carrying *tet*(B) represented only partial plasmids, sequences of the plasmids with highest identity were then used to search the wgs using BLASTn to identify other contigs carrying plasmid-related sequences.

**Table 1:** *Actinobacillus pleuropneumoniae* isolates carrying *tet*(B) resistance plasmids.

Isolate ID	Serovar	Location^a^	Year	MIC (μg/ml Tet)	Accession (wgs)	Contig in wgs^b^	Plasmid size^c^	Plasmid name	Most similar to
MIDG2567	8	Thirsk	2003	4	ERS134321	21, 71	3386^∗^	pM2567Tet	pTetHS016^d^
MIDG2650	8	Thirsk	2005	16	ERS134316	10	3366	pM2650Tet	pTetHS016
MIDG2656	2	Winchester	2005	16	ERS134610	2	3366^∗^	pM2656Tet	pTetHS016
MIDG2658	8	Thirsk	2005	16	ERS134322	15, 24	3376^∗^	pM2658Tet	pTetHS016
MIDG2661	8	Bury St Edmunds	2005	16	ERS134325	51	3366	pM2661Tet	pTetHS016
MIDG2666	8	Bury St Edmunds	2005	>16	ERS134330	4	3349	pM2666Tet	pTetHS016
MIDG3202	8	Bury St Edmunds	2006	16	ERS134333	2	3366^∗^	pM3202Tet	pTetHS016
MIDG3233	6	Bristol	2008	16	ERS134364	7, 20	3366^∗^	pM3233Tet	pTetHS016
MIDG3234	8	Starcross	2008	16	ERS134625	2, 109	3366	pM3234Tet	pTetHS016
MIDG3362	12	Thirsk	2008	8	ERS134382	7, 40, 111, 126	12666^∗^	pM3362MDR	pTetHS016 and pOV^e^
MIDG3379	8	Thirsk	2009	16	ERS134397	14, 124	3376	pM3379Tet	pTetHS016
MIDG3380	8	Thirsk	2009	16	ERS134636	50	3376	pM3380Tet	pTetHS016
MIDG3382	8	Thirsk	2009	8	ERS134400	15	3366	pM3382Tet	pTetHS016
MIDG3394	7	Thirsk	2010	8	ERS155334	16	3366	pM3394Tet	pTetHS016
MV780	8	Brazil	2009	≥16	JSVV00000000	44, 47, 53	5128^∗^	p780	pB1001^f^
MV460	8	Brazil	2007	≥16	JSVG00000000	48, 51, 54	5128	p460	pB1001
MV1022	8	Brazil	2011	≥16	JSVF00000000	45, 47, 51	5128	p1022	pB1001
MV5651	8	Brazil	2006	≥16	JSVY00000000	42, 45, 51	5128	p5651	pB1001

*^a^Other than the four Brazilian isolates (Pereira et al., 2015), specific locations are for previously described United Kingdom isolates (Bossé et al., 2017). ^b^Contig number(s) in draft wgs containing plasmid sequences. ^c^Sizes of isolated plasmids confirmed by sequencing are indicated (^∗^), otherwise sizes were predicted bioinformatically. ^d^pTetHS016 (accession KC818265) is a 3366 bp plasmid from a United Kingdom Haemophilus parasuis isolate (Luan et al., 2013). ^e^pOV (accession NC_019381) is a 13,551 bp plasmid from Pasteurella multocida. ^f^pB1001 (accession EU252517) is a 5128 bp plasmid from P. multocida in Spain.*

Plasmids were extracted from the *A. pleuropneumoniae* isolates MV780, MIDG2567, MIDG2656, MIDG2658, MIDG3202, MIDG3233, and MIDG3362 using the Qiaprep Spin Miniprep kit (Qiagen) according to the manufacturer’s protocol. The correct joining of contigs (i.e., ends of a single, or overlapping multiple contigs, as appropriate) was verified either by inverse PCR to amplify the region between the 5′ and 3′ ends of the *tet*(B) gene, or by amplification across the predicted contig junctions. The complete sequence of each extracted plasmid was confirmed using a primer walking strategy. Descriptions of all primers used in this study are shown in Table 2. The annotated sequences of plasmids p780, pM2567Tet, pM2656Tet, pM2658Tet, pM3202Tet, pM3233Tet, and pM3362MDR have been deposited in Genbank under the accession numbers MH457196 to MH457202, respectively.

**Table 2:** Primers used in this study.

Primer name	Sequence	Target/purpose
tetB_for	CGCATTGGTAATTACGTTATTCGATG	Amplification of an 1101 bp internal fragment of *tet*(B)
tetB_rev	GCTAAACCAATAATCCAAATCCAGC	
tetB_5′_out	CGTAATTACCAATGCGATCTTTGTC	Inverse PCR amplification and/or sequencing of the region between the ends of the *tet*(B) gene
tetB_3′_out	GTTAACCCCTCAAGCTCATGG	
rep_5′_out	TTGCCATAAGACTAGAGATTTCCTG	Sequencing out from 5′ end of *rep* in p780 (multiple priming sites in other plasmids)
rep_3′_out	TTTAAGAGGGGAATATGGCAACAC	Sequencing out from 3′ end of *rep* in plasmids other than p780 where multiple priming sites are present
3362_node40_out	TTGCCATAAGACTAGAGATTTCCTG	Confirmation of correct joining of contigs and sequencing of the pM3362MDR plasmid
3362_node111_out	AGCCCAAAAAGAGCCGATAGG	
3362_node126_out	TTAATGTTCAGCAGAGGGGAGG	
3362_rep_5′_out	TAGAACTCTCATTTCATCAAGCG	
3362_ISApl_5′_out	TCGTTGCACTTGGTTTGACAATTC	
3362_ISApl_3′_out	TGCCCTGTGCGAGTAAAATC	
780_rep_int_for	GGTTTTAGAGCCATCCATAACGG	Sequencing out from 3′ end of *rep* in p780
780_node44_out	GCCATTTTTACCTTCCTAATCTTCAG	Confirmation of correct joining of contigs and sequencing of the p780 plasmid

### Electroporation of Plasmids Into *Escherichia coli* MFD*pir*

Plasmids p780, pM2656Tet, and pM3362MDR were electroporated into the *E. coli* conjugal donor strain, MFD*pir* (Ferrieres et al., 2010). Transformants were selected on Brain Heart Infusion (BHI) agar supplemented with 0.3 mM diaminopimelic acid, containing 5 μg/ml tetracycline. The presence of plasmid in tetracycline resistant transformants was confirmed by PCR amplification using the *tetB*_for/*tetB*_rev primers (Table 2) prior to plasmid extraction, as above. Stability of the cloned plasmids in *E. coli* was assessed and compared to the endogenous plasmids in *A. pleuropneumoniae* as previously described (Moleres et al., 2015), with minor modifications. Briefly, following initial plating on selective agar, bacteria were cultured in 10 ml non-selective broth, which was serially passaged (1:100 dilution) eight times. Following the fourth and eighth passage, the stability of the plasmids was assessed by comparing the number of resistant cfu/ml on selective agar, to total cfu/ml on non-selective agar.

### Conjugal Transfer Experiments

In order to investigate mobilization from the *E. coli* MFD*pir* clones containing plasmids p780, pM2656Tet, and pM3362MDR, we used the same plasmid-free, tetracycline susceptible clinical *A. pleuropneumoniae* isolates as previously used to demonstrate conjugal transfer of ICE*Apl*1 and ICE*Apl*2 (Bossé et al., 2016; Li et al., 2018), namely MIDG3376 (serovar 6), MIDG2465 (serovar 7), MIDG3217 (serovar 8), and MIDG3347 (serovar 12). Matings were performed as previously described (Bossé et al., 2009), with selection on BHI-NAD containing 2.5 μg/ml tetracycline. The conjugation experiments were performed twice on independent occasions, with MIDG2331*ΔureC::nadV* [carrying *tet*(B) in ICE*Apl1*] as a positive control donor (Bossé et al., 2016).

## Results and Discussion

**FIGURE 1:**
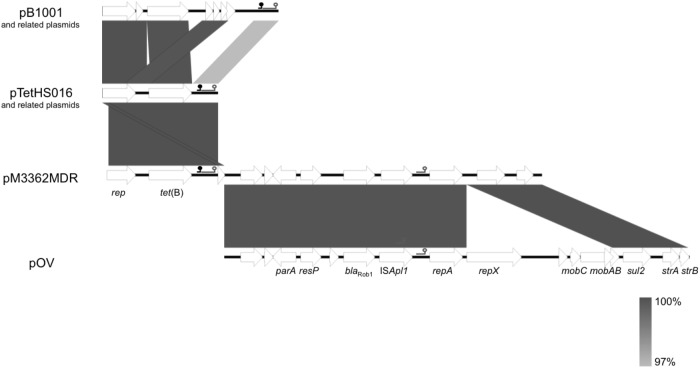
Schematic representation of the structure and organization of genes in different *Pasteurellaceae tet*(B) and multi-resistance plasmids. pB1001 from *Pasteurella multocida* and related plasmids from *Actinobacillus pleuropneumoniae* isolates (see Table 1); pTetHS016 from *Haemophilus parasuis* and related plasmids from *A. pleuropneumoniae* isolates (see Table 1); pM3362MDR from *A. pleuropneumoniae* isolate MIDG3362; pOV from *P. multocida*. Open reading frames are shown as arrows, with the arrowhead indicating the direction of transcription. Genes with known/predicted function are labeled [*rep*, *repA*, *repX*: plasmid replication; *tet*(B): tetracycline resistance; *parA*: plasmid partitioning; *resP*: predicted resolvase; *bla*_Rob-1_: β-lactam resistance; IS*Apl1*: transposase; *mobAB*, *mobC*: plasmid mobilization; *sul2*: sulfonamide resistance; *strA*, *strB*: streptomycin resistance]. The predicted origin of replication (*oriV*) is indicated by the symbol (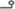) and the origin of transfer (*oriT*) by the symbol (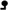) for each of the plasmids shown - note that pM3362MDR contains two different *oriV* sequences. Gray blocks between sequences indicate >97% nucleotide sequence identity according to the scale shown.

**FIGURE 2:**
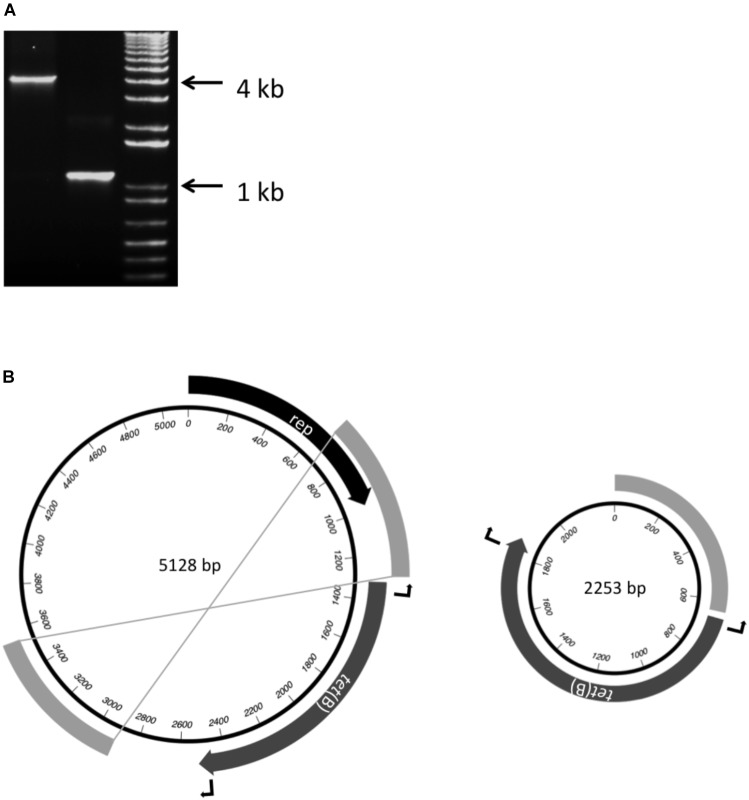
Detection of two different sized amplicons **(A)** produced by inverse-PCR from the ends of the *tet*(B) gene using DNA extracted from the *A. pleuropneumoniae* isolate MV780. **(B)** Schematic representation of p780 (5128 bp) and a smaller plasmid (2253 bp) formed by recombination between the direct repeat sequences (shown as unlabelled gray bands) flanking the *tet*(B) gene, as indicated by the crossed lines joining the ends of the repeat sequences in p780. The locations of the outward-facing primers at the ends of the *tet*(B) gene are indicated by the bent arrows, and lengths of sequence between the primers in the respective plasmids correspond to the correspond to the 4 and 1.1 kb amplicons detected in **(A)**.

In each of the four Brazilian isolates, a 1206 bp *tet*(B) gene was on a 1611 bp contig that shares 100% identity with the *rep* and *tet*(B) sequences of pB1001 (Figure 1), a 5128 bp plasmid from *Pasteurella multocida* (San Millan et al., 2009). The remaining sequences of this plasmid were found on two further contigs (644 and 2231 or 2233 bp) in each isolate, with the 644 bp contig (containing the last 329 bp of *rep*) matching sequences both upstream and downstream of *tet*(B) in pB1001. When inverse PCR was used to determine the length of the sequence flanking the *tet*(B) gene, two products (circa 4 and 1.1 kb, respectively; Figure 2A) were amplified, which when sequenced, indicated that the smaller amplicon was produced by deletion of 2875 bp mediated by recombination between the direct repeat sequences flanking *tet*(B) (Figure 2B). This type of instability has been reported in other plasmids (Dianov et al., 1991; Oliveira et al., 2010). It is unclear whether the smaller plasmid, lacking the full-length *rep* gene, is capable of stable replication.

In eight United Kingdom isolates, a single contig was found to contain the entire sequence of a plasmid almost identical to pTetHS016 (Figure 1; accession KC818265), a 3366 bp plasmid from a United Kingdom *Haemophilus parasuis* isolate (Luan et al., 2013), encoding a 1257 bp *tet*(B) gene along with a 978 bp *rep* gene. In each case, direct repeat sequences at either end of the contig were detected that allowed closure into circular plasmids of similar sizes (Table 1). A further five isolates had sequences similar to pTetHS016 divided over two contigs (Table 1). Inverse PCR produced similar sized amplicons (circa 2.2 kb) for sequences flanking the *tet*(B) gene in isolates with the pTetHS016-like sequences found on one or two contigs (Figure 1). Complete sequencing of pM2567Tet, pM2658Tet, pM3202Tet, and pM3233Tet confirmed the predicted sizes, with slight differences between plasmids seen in the intergenic regions.

Comparison of pB1001 and pTetHS016 (Figure 1) revealed that they share 95% identity over the majority of the smaller plasmid sequence, indicating that they likely evolved from the same origin. Differences around the 5′ and 3′ ends of *tet*(B) account for the variation in length of this gene between the plasmids, with a 110 bp deletion in the pB1001-type replicons resulting in a start codon 16 residues into the protein encoded by the larger genes, and a single base deletion at the 3′ end resulting in an alternate stop codon. The *tet*(B) gene from the larger pB1001-type plasmids (as well as 350 bp downstream, not present in the smaller pTetHS016-type), shows 100% identity with chromosomal and plasmid sequences from various species including *E. coli*, *Salmonella enterica*, and *Shigella dysenteriae* (e.g., accession numbers CP025254, CP022069, and CP026778), indicating a likely enterobacterial source, as has been shown for other resistance genes in *Pasteurellaceae* plasmids (Michael et al., 2018).

**FIGURE 3:**
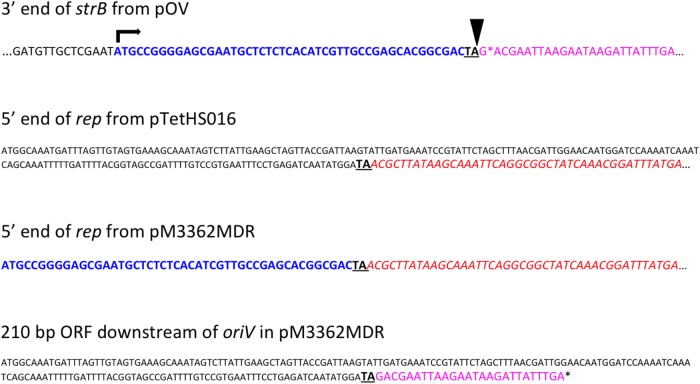
Schematic representation of the site of insertion of the pTetHS016 plasmid sequence into a pOV-like plasmid during formation of pM3326MDR. The stop codon (TAG) of the *strB* gene in pOV is followed by an asterisk (^∗^) indicating the end of the coding sequence. The insertion site of pTetHS016 into a pOV-like plasmid is indicated by a downward triangle. The TA dinucleotide present in the stop codon of *strB*, and that in the *rep* gene of pTetHS016, at which point the plasmids recombine, is shown in bold and underlined in each of the sequences shown. During recombination, the 5′ end of the pTetHS016 *rep* gene is displaced, forming the majority of the 210 bp ORF found downstream of the *oriV* in pM3362MDR. An alternate 5′ end for the *rep* gene in pM3362MDR is supplied from within the 3′ end of the disrupted *strB* gene (shown in blue text), whereas the stop codon of the 210 bp ORF in pM3362MDR is derived from sequence following the end of the *strB* gene (sequence shown in pink text). The majority of the *rep* gene in pM3362MDR is identical to that in pTetHS016 (sequence shown in red italic text).

In the genome of one United Kingdom isolate, MIDG3362, a 1257 bp *tet*(B) gene was identified by ResFinder on a 6496 bp contig, 3051 bp of which shares 100% identity with pTetHS016, and the remaining 3455 bp is 99% identical to sequences encoding *repA*, *sul2*, and *strA* found in pOV (accession NC_019381), a 13,551 bp plasmid from from *P. multocida*, and a related set of small multi-resistance plasmids from “*Actinobacillus porcitonsillarum*” (Matter et al., 2008) ranging in size from 8751 to 13,425 bp (accession numbers AJ830711, AM748705, AJ830712, and AM748706). These plasmids all further carry the β-lactamase gene, *bla*_Rob-1_. ResFinder results for MIDG3362 identified a 4820 bp contig carrying *bla*_Rob-1_, of which 4485 bp is 99% identical to sequences in pOV, and the remaining 336 bp is 100% identical with sequences in pTetHS016, with a 20 bp overlap, allowing the joining to pTetHS016 sequences on the *tet*(B) carrying contig. A further 1375 bp sharing 100% identity with sequences from pOV was found distributed over two additional contigs in the MIDG3362 genome. Overlapping sequences were identified between the ends of these two contigs, and those of the *tet*(B)- and the *bla*_Rob-1_-carrying contigs, allowing closure of a complete circular plasmid which was confirmed by PCR amplification and sequencing of products spanning the junctions. This 12666 bp plasmid, pM3362MDR, contains an almost complete copy of the pTetHS016 sequence (Figure 1). The pTetHS016 plasmid appears to have integrated seamlessly into the stop codon of the truncated *strB* gene in plasmid pOV by a single cross-over event at a TA dinucleotide (Figure 3), resulting in disruption of the 978 bp *rep* gene, such that the 5′ end of the gene is part of a 210 bp orf found downstream, whilst the majority of the *rep* gene is present as an 843 bp orf upstream of *tet*(B), having acquired an alternate start codon from within the 3′ end of the *strB* gene of pOV. There are no apparent homologous sequences in the *rep* and *strB* genes, thus integration appears to have been through illegitimate recombination. A 4255 bp segment of pOV, spanning from *repX* through the *mobCAB* genes, is not present in pM3362MDR, where only a 199 bp remnant of the *mobA* gene is present between *repA* and *sul2*. Deletion of this segment may explain why attempts to mobilize pM3362MDR from a conjugal donor strain into plasmid-free isolates of *A. pleuropneumoniae* were unsuccessful (see below).

Replicons which have evolved to be stably maintained in *Pasteurellaceae* frequently contain resistance genes that appear to have been acquired from enterobacterial plasmids (Ares-Arroyo et al., 2018; Michael et al., 2018). This is likely due to the fact that most *Pasteurellaceae* plasmids are capable of stable replication in *E. coli*, whereas the converse is rarely true, suggesting some differences in the origin (*oriV*) and/or proteins required for replication (Michael et al., 2018). In *Pasteurellaceae* species, many plasmids are ColE1-type replicons (related to, but distinct from those found in *Enterobacteriaceae*), which tend to carry the *mobCAB* mobilization genes, with the origin of transfer (*oriT*) as well as the *oriV* found upstream of *mobC* (Ares-Arroyo et al., 2018). Replication of ColE1-type plasmids relies on host factors; whereas plasmids carrying their own *rep* genes have cognate *oriV* sequences, typically AT-rich regions containing direct repeats (iterons) which specifically bind the Rep protein (Rajewska et al., 2012; Lilly and Camps, 2015; Wegrzyn et al., 2016). Both Rep-encoding and ColE1-type replicons have been described in *Pasteurellaceae* species, and some plasmids carry more than one *oriV*; for example, the pOV plasmid has both a ColE1 origin, and a RepA-specific *oriV* (discussed below).

**FIGURE 4:**
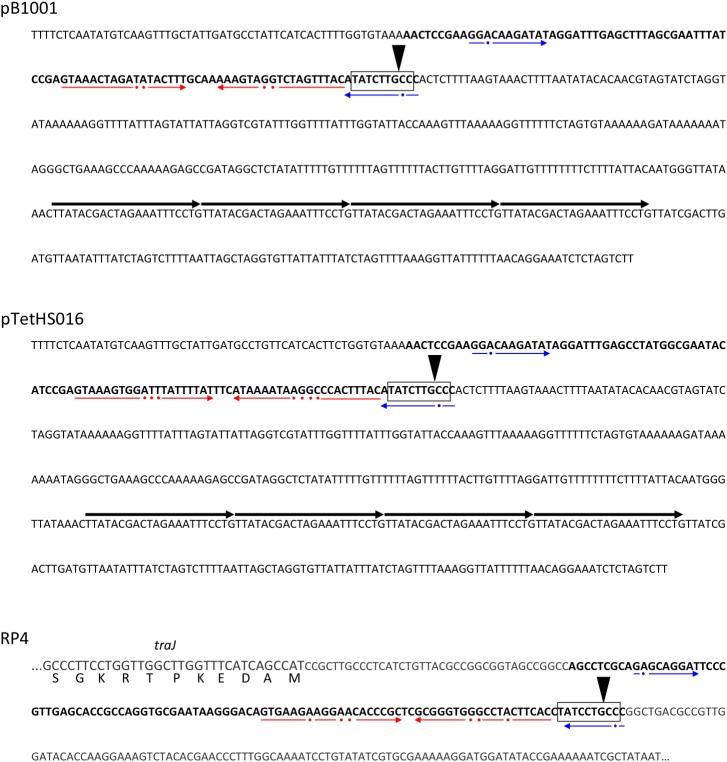
Nucleotide sequences from plasmids pB1001, pTetHS016, and RP4 indicating the respective origin of transfer (*oriT*) regions in bold text, with the *nic* recognition site boxed and the site of cleavage indicated by a downward triangle. The major imperfect inverted repeat sequences in each are underlined with red arrows (broken, with dots indicating non-conserved bases), and the sequences of a second imperfect inverted repeat underlined with blue arrows (broken, with dots indicating non-conserved bases). In pB1001 (and related *A. pleuropneumoniae* plasmids – see Table 1), the *oriT* is located immediately upstream of the predicted origin of replication (*oriV*), an AT-rich region containing four iterons (direct repeats of the sequence TTATACGACTAGAAATTTCCTG; indicated by the four contiguous arrows above the text) involved in binding of the Rep protein. In RP4, the *oriT* is located immediately upstream of the *traJ* gene sequence (for which only the 5′ end is shown encoded on the complement strand, with bases of the coding sequence in larger font, and with the respective amino acids shown below each codon). In RP4, the sequence downstream of the *oriT* does not contain the *oriV*, but rather leads to the divergently transcribed *traK* gene, following 225 bp of intergenic sequence.

The three different plasmids in this study, which were confirmed to stably replicate in both *A. pleuropneumoniae* and *E. coli* over eight passages in the absence of selection, have an AT-rich (74%) region of 438–458 bp containing four contiguous direct repeats (iterons) of the 22 bp sequence TTATACGACTAGAAATTTCCTG (shown for pB1001 and pTetHS016 in Figure 4). Although there is sequence variation between the AT-rich regions of the different plasmids, the iterons are identical, supporting that these are sites for binding the Rep protein encoded by all of these plasmids. In the smaller pB1001- and pTetHS016-type plasmids, the predicted replication origin immediately precedes the 978 bp *rep* gene, whereas in pM3362MDR, it is upstream of the 210 bp orf containing the 5′ end of the *rep* gene, as discussed above. It is unclear if the truncated 843 bp *rep* gene in pM3362MDR is functional, as there is a second predicted *oriV* located upstream of the *repA* gene derived from the pOV-like replicon, which may be responsible for replication of this plasmid (Figure 1). This 292 bp region has an AT-content of 69%, and contains four contiguous direct repeats of the 22 bp sequence TTAAAACCCTACAGATTTACGG, which is likely the iteron specific for binding of the RepA protein. In support of this, an identical *oriV* was previously described in other plasmids encoding the same Rep protein (Matter et al., 2008).

**FIGURE 5:**
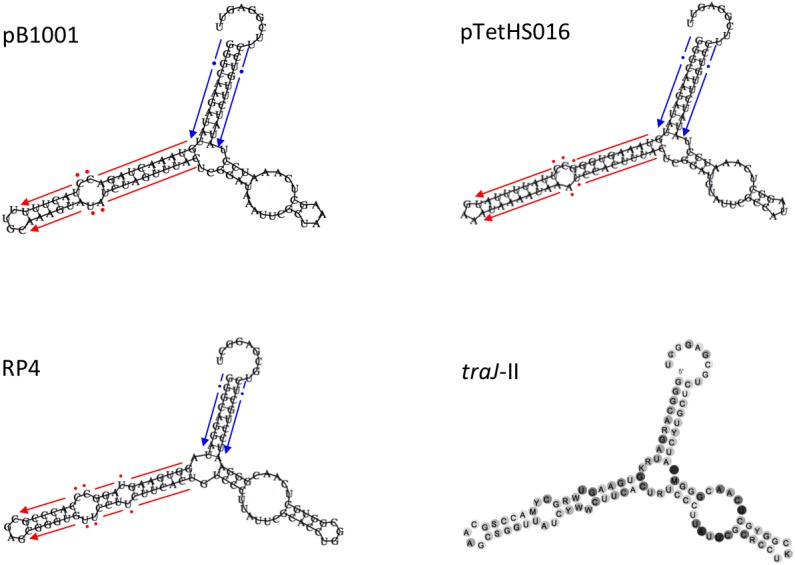
Schematic representation of the secondary structures of the *oriT* regions of plasmids pB1001, pTetHS016, and RP4, compared to the consensus structure of the non-coding RNA *traJ*-II found upstream of the *traJ* gene in various conjugative plasmids and chromosomal sequences (Rfam family RF01760). The red and blue arrows (broken, with dots indicating non-conserved bases) flanking the hairpins in the different *oriT* sequences correspond to the respective imperfect inverted repeat sequences shown in Figure 4.

Small mobilizable plasmids normally carry *mob* gene(s) encoding a relaxase (or multi-component relaxosome), which makes a single strand cut at the *nic* site of the *oriT* sequence, commonly found upstream (O’Brien et al., 2015). However, some mobilizable plasmids have been found that carry only a minimum *oriT*, requiring the relaxase to be supplied *in trans* (O’Brien et al., 2015). Relaxase proteins are specific for their cognate *oriT* sequences, and have been classified into six major families (i.e., MOB families C, F, H, P, V, and Q), which can be further divided into subfamilies (Garcillán-Barcia et al., 2009; Zrimec and Lapanje, 2018). Although the plasmids in this study do not encode known *mob* genes, they do all contain a 100–105 bp sequence (Figures 4, 5) located immediately upstream of the common *oriV*, containing regions of dyad symmetry that are characteristic of *oriT* sequences (Ziegelin et al., 1989; Pansegrau et al., 1990; O’Brien et al., 2015). These sequences are predicted to form secondary structures similar to the bioinformatically predicated sRNA *traJ*-II (*traJ*-II; Rfam family RF01760; Figure 5), found in the 5′ UTR of the *traJ* gene in various plasmid and chromosomal sequences (Weinberg et al., 2010). Indeed, the predicted *traJ*-II sequence in conjugative plasmid RK2/RP4 (accession number K00832.1) corresponds to that previously described as the *oriT* of this plasmid (Ziegelin et al., 1989; Pansegrau et al., 1990). Recently, a plasmid classification method was developed based on analysis of conserved structures in non-coding regions, which were shown to be highly conserved and discriminative for predicting the MOB family, even in the absence of encoded relaxases (Zrimec and Lapanje, 2018). Results obtained using this tool support our prediction that the *oriT* sequences identified in the plasmids in this study, like that of RP4, correspond to the MOBP family. However, as the largest family, MOBP includes numerous relaxases, some of which require accessory proteins for specific binding of their cognate *oriT* prior to cleavage of the *nic* site (Pansegrau and Lanka, 1991; Parker et al., 2005; Garcillán-Barcia et al., 2009; Zrimec and Lapanje, 2018).

In addition to having similar secondary structure, the *oriT* sequences of the plasmids in this study have predicted *nic* sites that share a high degree of identity with that of RP4, differing only at one base, however, the sequences of the major inverted repeat (found immediately upstream of the *nic* site) differ markedly (Figure 4). The right arm of this repeat region has been shown to provide specificity of the RP4 relaxosome via binding of the TraJ protein (Ziegelin et al., 1989; Pansegrau et al., 1990). We confirmed the inability of RP4-dependent conjugative transfer machinery, encoded by *E. coli* conjugal MFD*pir* (Ferrieres et al., 2010), to transfer representatives (pM2656Tet, pM3362MDR, and p780) of each of the three types of resistance plasmid in this study. Having been electroporated into MFD*pir*, the plasmids were shown to be capable of replication in this *E. coli* strain, as confirmed by PCR amplification of the encoded *tet*(B) gene from transformants, however, no transconjugants were obtained using any of the four plasmid-free *A. pleuropneumoniae* recipient strains. The control experiment demonstrated that these strains were successfully used as recipients for conjugal transfer of the tetracycline resistance integrative conjugative element, ICE*Apl1*, with conjugation frequencies of 10^−4^–10^−5^, both in this and the previous study (Bossé et al., 2016). It is possible that mobilization of the plasmids described in this study, homologs of which have been identified in different *Pasteurellaceae* species (suggesting horizontal transfer between them), requires specific conjugal transfer machinery that has yet to be identified. Various integrative and conjugative elements (ICEs) belonging to either the ICE*Hin1056* family (Mohd-Zain et al., 2004; Bossé et al., 2016), the SXT/R391 family (Li et al., 2018; Xu et al., 2018), or a set related ICEs including ICE*Pmu1* and ICE*Mh1* (Michael et al., 2012; Eidam et al., 2015), are present in different *Pasteurellaceae* species, but none have sequences similar to the predicted plasmid *oriT*s (or their major inverted repeats), suggesting the ICE-encoded relaxases would not likely be capable of mobilizing these plasmids.

In summary, we have identified and characterized three related tetracycline resistance plasmids circulating in isolates of *A. pleuropneumoniae* in the United Kingdom and Brazil. Two are almost identical to plasmids pTetHS016 and pB1001, found in other members of the *Pasteurellaceae* (i.e., *H. parasuis* and *P. multocida*, respectively), that carry only the *tet*(B) and *rep* genes. The third, pM3362MDR, is a novel multi-resistance plasmid, carrying *bla*_Rob-1_, *sul2*, and *strA* in addition to *tet*(B), and appears to have been derived from insertion of the pTetHS016 plasmid into pOV (from *P. multocida*), or a related plasmid. Co-existence of multiple plasmids in the same isolate has previously been reported for *P. multocida* and *A. pleuropneumoniae* (San Millan et al., 2009; Bossé et al., 2015), providing the opportunity for either homologous or illegitimate recombination. The presence of (nearly) identical plasmids in multiple *Pasteurellaceae* suggests horizontal transfer between these different species, which can share the same niche in the respiratory tract of pigs. Although a putative MOBP-related *oriT* was identified in all plasmids, we were unable to demonstrate mobilization using an *E. coli* donor strain expressing conjugation machinery specific for RP4-type plasmids. It is possible that these plasmids could be mobilized by cognate conjugation machinery, perhaps *Pasteurellaceae*-specific, which remains to be identified.

## Author Contributions

JB, PL, SW, GS, and DB conceived the study. SW provided UK clinical isolates. YHL, GS, JB, YWL, and CR produced the data. JB, YHL, GS, and RF analyzed the data. JB and YHL wrote the paper.

## Conflict of Interest Statement

The authors declare that the research was conducted in the absence of any commercial or financial relationships that could be construed as a potential conflict of interest.
